# A novel pyramid temporal causal network for weather prediction

**DOI:** 10.3389/fpls.2023.1143677

**Published:** 2023-03-31

**Authors:** Minglei Yuan

**Affiliations:** Anhui University of Finance and Economics, Bengbu, China

**Keywords:** temporal convolutional networks (TCN), weather forecasting, deep learning, time serial model, loss function

## Abstract

In the field of deep learning, sequence prediction methods have been proposed to address the weather prediction issue by using discrete weather data over a period of time to predict future weather. However, extracting and utilizing feature information of different time scales from historical meteorological data for weather prediction remains a challenge. In this paper, we propose a novel model called the Pyramid Temporal Causal Network (PTCN), which consists of a stack of multiple causal dilated blocks that can utilize multi-scale temporal features. By collecting features from all the causal dilated blocks, PTCN can utilize feature information of different time scales. We evaluate PTCN on the Weather Forecasting Dataset 2018 (WFD2018) and show that it benefits from multi-scale features. Additionally, we propose a multivariate loss function (MVLoss) for multivariate prediction. The MVLoss is able to accurately fit small variance variables, unlike the mean square error (MSE) loss function. Experiments on multiple prediction tasks demonstrate that the proposed MVLoss not only significantly improves the prediction accuracy of small variance variables, but also improves the average prediction accuracy of the model.

## 1 Introduction

Making accurate weather predictions has always been a challenging task in human history. Currently, the main methods for weather forecasting involve using large supercomputers to simulate atmospheric movement. The Weather Research and Forecasting model (WRF) is the most popular method for both research and real-time weather forecasting worldwide, and its forecasting accuracy has continuously improved with technological development. However, existing methods rarely use historical data to infer future weather, and not all periodic characteristics of meteorology can be accurately defined.

In recent years, deep learning-based sequence prediction models, such as Recurrent Neural Networks (RNNs), Long Short Term Memory (LSTM), and Gated Recurrent Units (GRU), have been proposed. However, these models still suffer from gradient vanishing or explosion while learning long sequences. To overcome this problem, various methods, including WaveNet and Temporal Convolutional Network (TCN), have been proposed. However, these methods only use features of the last layer for prediction and do not fully utilize features from different time scales, which is important in weather forecasting.

To address these issues, we propose a novel model called Pyramid Temporal Causal Network (PTCN) for accurate weather predictions. The feature extracting network of PTCN is constructed using dilated causal convolutional layers and shortcut connections, similar to TCN. We also introduce a pyramid-like structure in PTCN to collect and merge features extracted from different layers of the feature extracting network, which have different receptive fields along the time axis. This provides a multi-scale feature representation that helps the model learn the weather’s internal rules at different time scales.

We also propose a new loss function for multivariate prediction that considers the fluctuation range of different variables. When using mean square error (MSE) as the loss function for multivariate regression, small variance variables’ predicted results are often unsatisfactory. Therefore, we propose a multivariate loss function that takes into account the variance of all the predicted attributes, in order to help the model generate feasible predictions for all attributes.

This work has two main contributions:

1. To the best of our knowledge, this is the first work to model different time-scale features in sequence prediction problems, especially for weather predictions. We evaluate the proposed model on a large-scale Weather Forecasting Dataset 2018 (WFD2018) and show that PTCN achieves higher prediction accuracy compared to some commonly used deep learning-based time series forecasting methods and WRF models.
2. We propose a loss function for multivariate regression that considers the fluctuation range of different variables, and experimental results show that it significantly improves the average accuracy of multivariate weather prediction tasks.

## 2 Related work

### 2.1 Time series prediction models

Temporal Convolutional Network (TCN) Bai et al. (2018) is an effective temporal convolutional structure for sequence modeling problems. TCN integrates causal convolution, dilated convolution, and shortcut connections to gain a large receptive field with few layers, making it powerful for sequence prediction tasks. Recent works have shown TCN’s great potential in various time series tasks, including 3D motion prediction and speech separation Kanazawa et al. (2019); Luo and Mesgarani (2019). Due to its properties and suitability for our task, TCN-like blocks are designed in our network to perform weather forecasting

In addition to TCN, there are other related works in the field of time series prediction that have been proposed recently. One such work is the Transformer-based architecture, which has been successful in natural language processing and has been applied to time series prediction tasks Vaswani et al. (2017). Another related work is the Graph Convolutional Network (GCN) Wu et al. (2021), which is suitable for modeling complex relationships among variables and has been used for multivariate time series prediction Lipton et al. (2015); Wu et al. (2021). Another popular approach is Recurrent Neural Networks (RNNs) Williams and Zipser (1989), which have been widely used for time series prediction. One recent work is the application of RNNs for solar energy prediction Elsaraiti and Merabet (2022).

Other recent works have explored the use of ensemble models for time series prediction. One such work is the use of Convolutional Neural Networks (CNNs) and RNNs in an ensemble model for electricity load forecasting Mansouri and Akbari (2014). Another work proposed a deep learning ensemble model combining various architectures, including TCN and RNNs, for stock price prediction Srivinay et al. (2022).

While there are several works in the field of time series prediction, including TCN, that use deep learning models, PTCN is different from these approaches in several ways. One major difference is the use of a pyramid structure to combine features from different scales, which is inspired by FPN. While TCN also uses dilated convolution to increase the receptive field, PTCN uses a combination of dilated and causal convolutions. Additionally, PTCN incorporates a novel feature extraction block that includes a temporal attention mechanism and a residual block.

### 2.2 Feature extraction and attention mechanisms in time series prediction

Feature Pyramid Network (FPN) Lin et al. (2017) is a method for fully utilizing multiple levels of feature maps generated by deep neural networks. This approach was proposed by Lin et al. in 2017 and achieved state-of-the-art performance in the COCO 2016 challenge Lin et al. (2014) for multi-scale object detection. Many other image detection or segmentation approaches also take advantage of information from different layers of the ConvNet. For example, Fully Convolutional Networks (FCN) sum up partial scores calculated on multiple scales to derive the final score of each category for semantic segmentation problems. The prediction of Single Shot Detector (SSD) Liu et al. (2016) is based on using default boxes in 6 different layers of its backbone network. MSDNet Huang et al. (2017) maintains coarse and fine level features throughout the network, using fine level features from previous layers and coarse level features to generate the next layer’s features. The idea of combining features from different TCN layers in our proposed Pyramid Temporal Causal Network (PTCN) is inspired by FPN, which utilizes information from different scales to make predictions.

In addition to FPN, there are several recent works in the field of feature extraction that have been proposed. One such work is the Squeeze-and-Excitation (SE) Network Hu et al. (2018), which adaptively recalibrates channel-wise feature responses by explicitly modeling interdependencies between channels. Another 91 related work is the Attention Mechanism Bahdanau et al. (2015), which has been widely used in natural language processing and computer vision to selectively attend to different parts of the input. Attention Mechanism can also be used in the context of time series prediction to focus on important time steps or variables.

Recently, there have been several works that combine deep learning with traditional time series analysis methods. One such work is the use of deep learning and Fourier analysis for wind speed prediction Liu et al. (2018). Another work proposed a deep learning approach that combines Long Short-Term Memory (LSTM) and Wavelet Transform for temperature prediction Zolfaghari and Golabi (2021). Additionally, a hybrid model based on the Ensemble Empirical Mode Decomposition (EEMD) and LSTM was proposed for wind speed and direction prediction Qu et al. (2016).

Finally, there have been several works that explore the use of deep learning for weather forecasting. One recent work proposed a deep learning model that incorporates a physical model for short-term rainfall forecasting Qiu et al. (2017). Another work applied deep learning models to predict maximum daily temperature and precipitation Feng et al. (2019)

Regarding feature extraction, while FPN and other approaches focus on image processing, PTCN’s feature extraction is designed specifically for time series data. While attention mechanisms have been used in natural language processing and image processing, PTCN’s temporal attention mechanism is designed to attend to important time steps in a time series.

## 3 Weather prediction preliminaries

We approach weather prediction as a sequence prediction problem, formally expressed as follows: given a sequence of historical values *h*_1_,⋯,*h*_i_,⋯,*h*_t_ at different time steps, where *h_i_* indicates the historical weather information at time step *i* and contains meteorological factors, we aim to predict the values Ŷ*t*+1,⋯,Ŷ*t*+*p*. The problem is to learn the mapping function *F*(·) as defined in Equation 1.


(1) $$F(h_{1},\cdots ,h_{i},\cdots ,h_{t})=\hat{Y}t+1,\cdots ,\hat{Y}t+p$$

Our objective is to find a model that minimizes the expected loss between the predicted outputs and the actual outputs *Y_t+_*_1_,⋯,*Y_t+p_*, as defined in Equation 2.


(2) $$Loss=L(Y_{t+1},\cdots ,Y_{t+p},F(h_{1},\cdots ,h_{t}))$$

There are some of the difficulties associated with predicting weather using deep learning-based methods. These difficulties are as follows:

Non-linear Relationships: The relationship between weather variables is complex and nonlinear, making it challenging to model accurately using traditional statistical methods.

Temporal Dependencies: Weather patterns exhibit temporal dependencies, which means that current weather conditions are dependent on past weather conditions. This makes it essential to incorporate temporal dependencies into the model to accurately predict future weather patterns.

Extreme Weather Events: Extreme weather events, such as hurricanes, tornadoes, and heatwaves, are challenging to predict accurately, even with deep learning-based methods. This is because these events are rare and have complex dynamics, which are difficult to model accurately.

Addressing these challenges is essential for developing accurate deep learning-based weather prediction models. The PTCN model proposed in this paper addresses some of these challenges by incorporating multi-scale historical data and using causal convolutions to capture temporal dependencies.

## 4 The pyramid temporal causal network

**Figure 1:**
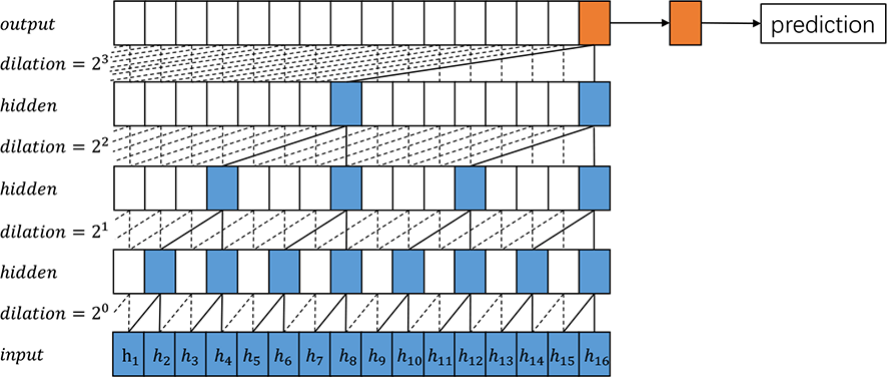
An overview of the proposed TCN architecture.

**Figure 2:**
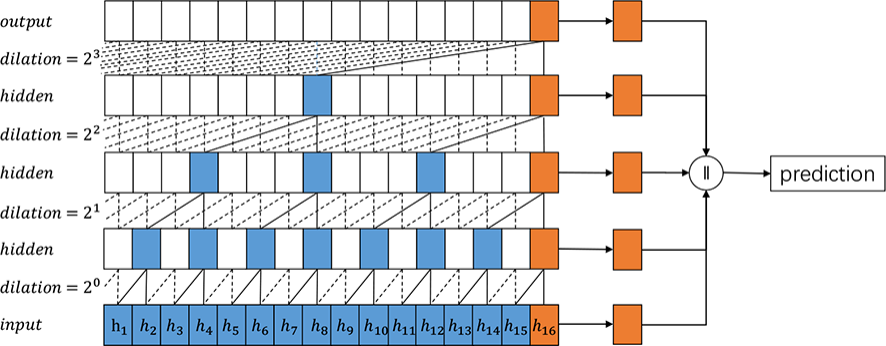
An overview of the proposed Pyramid TCN architecture.

In order to improve the performance of TCN, we propose the Pyramid Temporal Causal Network (PTCN), which utilizes information on different time scales to achieve a more accurate time sequence prediction. With the help of the information in different layers, PTCN can make use of the information flow of various time scales and reuse previous features. While similar to TCN, PTCN is distinct and designed to obtain different time scale features. **Figures 1**, **2** provide an overview of how TCN and PTCN work for time series prediction. The PTCN model utilizes multiple scales of historical data to capture both short-term and long-term patterns of weather changes. The hourly historical weather data is used to capture the short-term temporal dependencies of weather conditions, while the daily historical data are used to capture the daily patterns and long-term trends of weather changes. The PTCN model employs a pyramid structure to integrate these multiple scales of information. Specifically, the hourly data is fed into the bottom of the pyramid, while the daily data are fed into the middle and top of the pyramid, respectively. The model then uses causal convolutions to learn the temporal dependencies among these different scales of data, and generates the final weather prediction output.

We discuss the details on causal dilated convolution, causal dilated block, prediction module with pyramid temporal features, and multivariate loss function in the following subsections.

### 4.1 Causal dilated convolution

The Pyramid Temporal Causal Network (PTCN) model consists of several components that work together to capture multi-scale information and improve the accuracy of weather forecasting. One of the key components of the PTCN model is the causal dilated block, which is used to learn the temporal dependencies between different time scales.

The causal dilated block is composed of causal dilated convolutions, as described in Section 4.1. The output of each causal dilated block is fed into a residual connection, which allows the model to learn residual features and avoid the vanishing gradient problem. The output of the residual connection is passed through a ReLU activation function and a batch normalization layer before being fed into the next causal dilated block.

The use of causal dilated convolution allows the model to learn long historical sequences using a small number of layers. The receptive field of the causal dilated convolution can be calculated using Equation 3. The number of layers for the causal dilated convolution is determined according to Equation 4.


(3) $$r=\left(k-1\right)\times {\left(d\right)}^{\ell }$$

(4) $$L=\left\lceil log_{k}^{t}\right\rceil$$

Here, *k*, *d*, ℓ, and *t* represent the kernel size, dilation rate, layer index, and input sequence length, respectively. The receptive field of the last channel in the output layer can cover the entire input sequence under these conditions.

Causal dilated convolution is formalized in Equation 5.


(5) $$x_{out}=CausalDilated\left(x_{in},d,k\right)$$

Here, *x_in_* and *x_out_* indicate the input and output of the causal dilated convolution, respectively. *d* refers to the dilated rate, and *k* refers to the kernel size of the causal convolutional layer.

### 4.2 Causal dilated block

The causal dilated block is comprised of a branch of transformations *F* and an identity mapping function *I*. Each causal dilated block forms one layer of PTCN. We formalize the causal dilated block as follows:


(6) $$H_{i+1}=\sigma \left(F\left(H_{i}\right)+I\left(H_{i}\right)\right)$$

where *F* and *I* denote the residual mapping and identity mapping functions, respectively, and σ(·) represents an activation function. *H_i_* is the input and *H_i_*_+1_ is the output of the *i*-th causal dilated block (layer) in PTCN.

**Figure 3:**
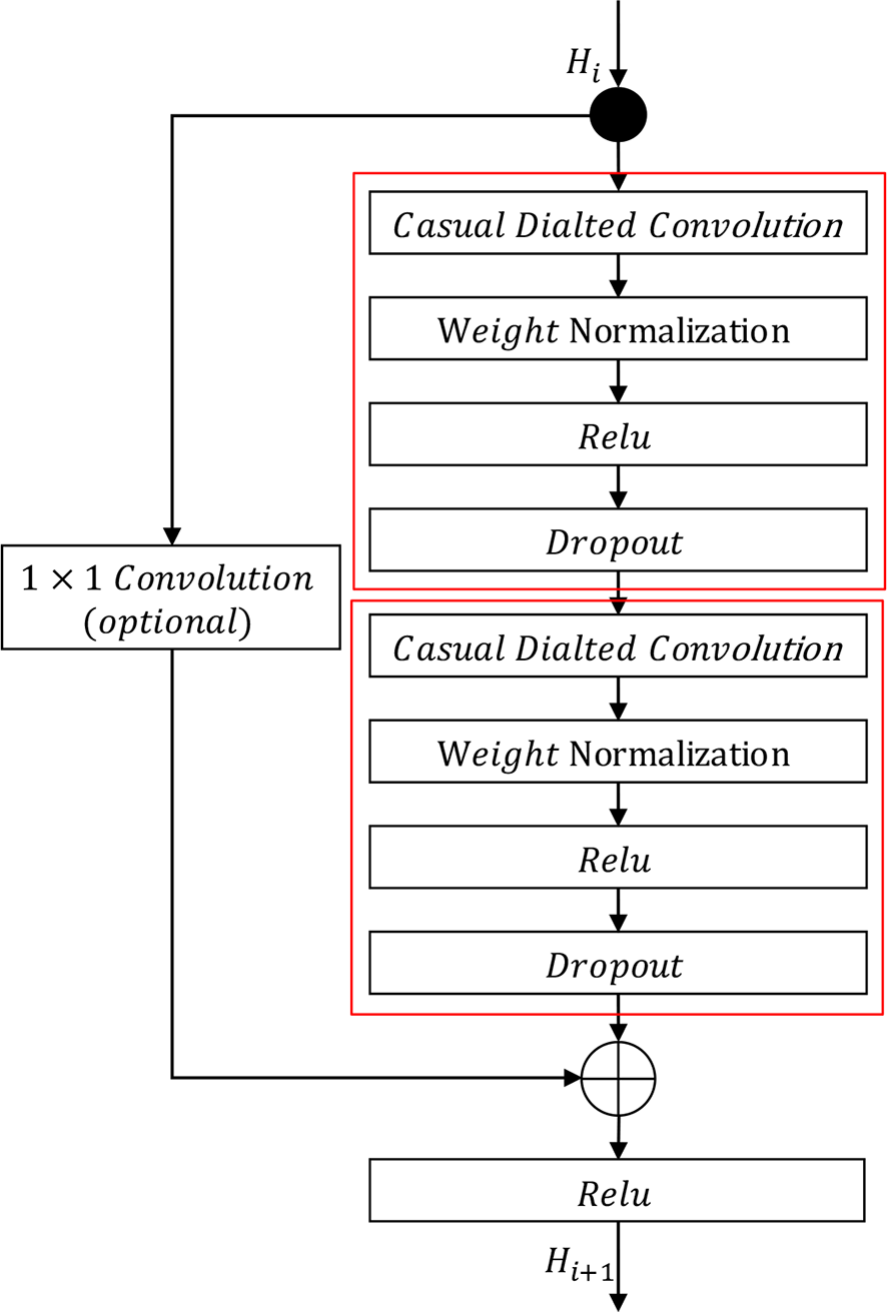
Causal dilated block. The residual mapping function is represented by the two red boxes, and the identity mapping function is on the left. The symbol ⊕ denotes element-wise addition.


The residual mapping function and identity mapping function are depicted in **Figure 3**. The residual mapping function consists of causal dilated convolution, weight normalization, non-linear activation functions, and dropout. We employ weight normalization proposed by? on causal dilated convolution, use ReLU Glorot et al. (2011) as a non-linear activation function, and adopt a dropout function proposed by Hinton et al. (2012) to mitigate overfitting. Furthermore, to expand the receptive field, we set the dilation rate of each causal dilated convolution according to the layer in which the causal dilated block is located. In this paper, we set the dilation rate as per Equation 7.

(7) $$d=2^{\ell }$$

where ℓ denotes the layer of the causal dilated block.

The identity mapping function is implemented using a shortcut connection similar to standard ResNet He et al. (2016). If the output *H_i_*_+1_ of the residual mapping function has the same dimensions as the input *H_i_*, *H_i_* is directly added to *H_i_*_+1_ by element-wise addition. Otherwise, *H_i_* is transformed into the same dimension as *H_i_*_+1_ using a 1×1 convolution before being added to *H_i_*_+1_.

### 4.3 Prediction module with pyramid temporal features

The prediction module is comprised of two parts: a multi-scale feature collector and a multivariate predictor.

The multi-scale feature collector selects features from all the causal dilated blocks in different layers. Specifically, only the features of the last time step of each causal dilated block (or layer) are saved, as shown in **Figure 2**. The features selected from different causal dilated blocks imply information of different time scales. We only select features from the last time step of each causal dilated block as the most recent information has a more significant impact on the future. Moreover, this is a trade-off between computational efficiency and prediction accuracy.

Multi-scale information plays a crucial role in improving the accuracy of weather forecasting. Weather patterns exhibit different temporal scales, ranging from hourly changes to seasonal variations. Each of these temporal scales has its unique characteristics and requires different methods of analysis to capture the patterns and trends accurately. By utilizing multi-scale historical data, the PTCN model can capture the different temporal patterns and trends in weather data, and integrate them effectively to produce more accurate weather predictions. The hourly data captures the short-term dynamics and dependencies of weather changes, such as temperature fluctuations, wind speed, and precipitation. Daily data captures the daily patterns, such as the daily temperature cycle, the probability of rainfall, and the intensity of wind. The monthly data captures the long-term trends and seasonal variations, such as seasonal temperature changes, monsoon patterns, and extreme weather events.

Integrating these multiple scales of information helps to reduce the impact of uncertainties, noise, and errors that are inherent in individual data scales. The PTCN model’s pyramid structure, which uses causal convolutions to learn the temporal dependencies between the different scales of data, further enhances the model’s ability to capture the complex temporal patterns and trends in weather data. In summary, by utilizing multi-scale information, the PTCN model can capture the different temporal patterns and trends in weather data and integrate them effectively to produce more accurate weather predictions. This approach helps to improve the reliability and robustness of weather forecasting, which is crucial for many applications, including agriculture, transportation, and disaster management.

The output of the multi-scale feature collector is fed into a multivariate predictor, which predicts the future values of all variables simultaneously. The multivariate predictor consists of several fully connected layers and a Softmax activation function. The output of the Softmax activation function represents the predicted values of all variables at the next time step.

The multivariate predictor is a linear transformation function designed to transform the data collected from the multi-scale feature collector into predicted values. In practice, the model needs to predict p*q variables, where *p* refers to the length of time and *q* refers to the type of variable to be predicted.

**Table 1** shows the comparison between the proposed PTCN model and other models in terms of the number of parameters, FLOPs (Floating Point Operations), and prediction accuracy. The results demonstrate that the PTCN32+ model achieves better performance in terms of the AA index, while only slightly increasing the number of parameters and FLOPs compared to TCN32 model.

**Table 1:** Floating point operations (FLOPs), number of parameters, and average accuracy (AA) of the proposed and comparison models.

Index	FLOPs	parameters	AA
LED	414.97M	52.04K	69.57%
GRU	312.67M	39.11K	71.98%
LEDA	2.04G	60.42K	71.33%
TCN16	429.44M	13.19K	75.96%
PTCN16	430.18M	16.07K	77.65%
TCN16+	429.44M	13.19K	77.96%
PTCN16+	430.18M	16.07K	78.42%
TCN32	1.66G	48.84K	76.63%
PTCN32	1.66G	54.60K	78.44%
TCN32+	1.66G	48.84K	78.13%
PTCN32+	1.66G	54.60K	79.04%

All experimental results are based on 64 hours of historical data to predict the next 12 hours.

For example, compared to the TCN32 model, the proposed PTCN32+ model achieves a 2.41% improvement in the AA index while only increasing the number of parameters by 5.8K. Similarly, compared to the LEDA model, the proposed PTCN32+ model achieves a 9.47% improvement in the AA index while increasing the number of parameters and FLOPs by 2.56K and 1245M, respectively.

These results suggest that the proposed PTCN32+ model can significantly improve the prediction accuracy while only slightly increasing the computational complexity. Therefore, it can be considered as an efficient and effective solution for multivariate time series prediction tasks.

### 4.4 Multivariate loss function

When predicting multivariate variables using the proposed sequence-to-sequence model, the prediction model has different preferences for variables with different variances. In practice, variables with high variance affect the model more, resulting in lower prediction accuracy for small variance variables. To address this, we propose a multivariate loss function (MVLoss). We calculate the mean and global variance of each attribute to be predicted, as shown in Eq. (8) and Eq. (9), and define the multivariate loss function as shown in Eq. (10):


(8) $${\bar{y}}^{j}=\sum_{i=1}^{n}y_{i}^{j}$$

(9) $$\mathrm{var}(y^{j})=\frac{\sum_{i=1}^{n}{\left(y_{i}^{j}-{\bar{y}}^{j}\right)}^{2}}{n}$$

(10) $$L(\hat{Y},\text{Y})=\sum_{j=1}^{q}\frac{\sum_{i=1}^{p}{\left({\hat{y}}_{i}^{j}-y_{i}^{j}\right)}^{2}}{p\times q\times \mathrm{var}(y^{j})}$$

Here, *n* represents the number of data points in the training dataset, *L*(·) is the proposed MVLoss, *p* represents the length of the predicted sequence, *q* represents the number of attributes that need to be predicted, ŷ represents the predicted value, *y* represents the observed value, and $y_{i}^{j}$ represents the true value of the *j*-th attribute at the *i*-th time step.

In practice, we use a combination of mean squared error (MSE) loss and MVLoss to optimize the model, as shown in Eq. (12). The MSE is detailed in Eq. (11). Moreover, MVLoss mentioned in this paper all refer to Eq. (12):


(11) $$MSE(\hat{Y},Y)=\frac{\sum_{j=1}^{q}\sum_{i=1}^{p}{\left({\hat{y}}_{i}^{j}-y_{i}^{j}\right)}^{2}}{p\times q}$$

(12) $$LOSS(\hat{Y},Y)=\lambda_{1}MSE(\hat{Y},Y)+\lambda_{2}L'(\hat{Y},Y)$$

Here, λ_1_ and λ_2_ are hyperparameters that control the relative contribution of the two loss functions to the overall loss.

## 5 Experiments and discussions

We present the experimental results for three tasks in this section, which involve predicting weather conditions for the next 6, 12, and 24 hours based on 32, 64, and 128 hours of historical weather information. The predicted variables include: (1) 2-meters temperature (t2m); (2) 2-meters relative humidity (rh2m); and (3) 10-meters wind speed (w10m).

### 5.1 Dataset

We conduct our experiments using the Weather Forecasting Dataset 2018 (WFD2018), a benchmark dataset provided by the AI Challenger Global AI Contest aic (AI Challenger, 2018). This dataset contains meteorological data collected through multi-station, multi-element, long-sequence, and high-time-density observations. It includes data from 10 stations and contains atmospheric pressure, temperature at the height of 2 meters, humidity at the height of 2 meters, wind speed at the height of 10 meters, and other meteorological information. The data are automatically collected by sensors every hour.

The training set covers 1188 days from March 1st, 2015 to May 31st, 2018, the validation set consists of 89 days from June 1st, 2018 to August 18st, 2018, and the test set includes 27 days from August 29th, 2018 to September 24th, 2018. In the training dataset, missing values are filled with valid data from either the front or the back.

### 5.2 Comparison methods

To demonstrate the effectiveness of the proposed method, we compared it with five state-of-the-art models on the WFD2018 dataset. These comparison methods are described below:

- WRF: Weather Research and Forecasting model (WRF) is a widely used method for weather forecasting, which leverages actual atmospheric conditions or idealized conditions data collected by weather satellites wrf (UNC-Chapel Hill, 2016). The prediction values of WRF are released by Beijing Meteorological Bureau, China aic (AI Challenger, 2018; Beijing Weather Bureau, 2019).
- GBR: Gradient Boosting Regressor (GBR) is an ensemble learning method that is suitable for regression prediction tasks and has a strong generalization ability Ye et al. (2009). In our experiment, we use 100 base estimators with a depth of 5.
- LED: Long Short-Term Memory (LSTM) Encoder-Decoder (LED) model is suitable for sequence prediction tasks. The LSTM Encoder-Decoder model consists of two parts: an Encoder that encodes the input sequence into a fixed vector representation, and a Decoder that parses vectors to a target sequence Cho et al. (2014).
- LEDA: LSTM Encoder-Decoder model with an attention mechanism (LEDA) can overcome the problem of converting the input sequence into a fixed-length vector Bahdanau et al. (2015).
- TCN: Temporal Convolutional Networks (TCN) incorporate a simple convolutional neural network architecture that can be used for sequence modeling. TCN can capture timing dependencies as it combines causal convolution, residual connection, and dilation convolution. Additionally, Bai et al. (2018) reported that TCN usually has better performance than LSTM and GBR when these models have parameters of similar size.

The following approaches are the proposed models:

- PTCN: Pyramid Temporal Convolutional Networks (PTCN) uses the same configuration as TCN, and the only difference is that PTCN uses a multi-scale feature representation from different causal dilated blocks to predict weather results.
- TCN+: TCN+ is the same as TCN, except that the loss function uses our proposed MVLoss.
- PTCN+: PTCN+ is the same as PTCN, except that the loss function uses our proposed MVLoss. In our experiment, Λ_2_ uses the value of 1, and Λ_2_ uses the value of 50, which is determined based on experimental results.

Note that LED, LEDA, TCN, and PTCN are all trained with MSE loss function. Also, we use a number used in the model names refers to the number of channels used in the hidden layer of the corresponding TCN or PTCN model. For example, PTCN32 refers to a PTCN model with 32 channels in the hidden layer, while PTCN16 refers to a PTCN model with 16 channels in the hidden layer.

### 5.3 Parameter settings

We preprocessed the training, validation, and testing sets using batch normalization. The batch size was set to 256, and we used the Adam optimizer Sutskever et al. (2013) for training. The learning rate was initially set to 0.01 and reduced by a factor of 10 every 30 epochs. We trained all deep learning models for 200 epochs.

For the TCN and PTCN models, we initialized the weights of the one-dimensional convolutions and linear transformations using the weight normalization method Salimans and Kingma (2016). We used ReLU Glorot et al. (2011) as the default activation function and applied a dropout rate of 0.05 after all causal dilated convolutions, which randomly sets some of the input tensor elements to zero.

The LED and LEDA models have the same basic structure and parameters, including LSTM models with 64 hidden dimensions, one layer, and a bidirectional setting of False.

## 6 Experiments and discussions

### 6.1 Evaluation metrics

We use five indicators to evaluate the forecast results, including Root Mean Square Error (***RMSE***) Plutowski et al. (1996), Mean Absolute Percentage Error (***MAPE***) Armstrong and Collopy (1992), coefficient of determination (**R^2^**) (Rodgers and Nicewander, 1988), ***accuracy***, and Average Accuracy (***AA***). RMSE evaluates the extent to which data deviates from the ground truth. MAPE can measure forecast accuracy in the trend forecasting method. A smaller value of RMSE or MAPE indicates better performance. *R* ^2^ is a common metric for evaluating the merit of a regression model, which measures the linear correlation between variables. The closer *R* ^2^ is to 1, the better the performance of the model. Accuracy is a widely used performance metric for classification problems, and it indicates how often the model’s predictions are correct. The accuracy score is calculated by dividing the number of correct predictions made by the model by the total number of predictions, and it is expressed as a percentage. It is a simple and intuitive metric that provides a quick understanding of the overall performance of the model. We convert the results of meteorological predictions into accuracy using Equation 16. The accuracy metric used in our paper is derived from the mean absolute error (MAE) between the predicted and actual values, and that it represents the percentage of predictions that are within a certain tolerance range of the actual values. The greater the accuracy, the better the performance of the model. AA is the average of the predictive accuracy of the three variables rh2m, t2m, and w10m. AA indicates the overall performance of the model when predicting multiple variables.

The mathematical forms of RMSE, MAPE, *R* ^2^, and accuracy are defined as Equations 13, 14, 15, and 16, respectively:


(13) $$RMSE=\sqrt{\frac{1}{n}\sum_{i=1}^{n}{\left(y_{i},{\hat{y}}_{i}\right)}^{2}}$$

(14) $$MAPE=\frac{10^{2}}{n}\cdot \sum_{i=1}^{n}\cdot \cdot \left|\frac{y_{i}-{\hat{y}}_{i}}{y_{i}}\right|$$

(15) $$R^{2}=1-\frac{\sum_{i=1}^{n}{\left(y_{i}-{\hat{y}}_{i}\right)}^{2}}{\sum_{i=1}^{n}{\left(y_{i}-\bar{y}\right)}^{2}}$$

(16) $$accuracy=1-\frac{\sum_{i=1}^{n}\left|y_{i},{\hat{y}}_{i}\right|}{\sum_{i=1}^{n}\left|y_{i}\right|}$$

Where ${\hat{y}}_{í}$ , *y_i_*, and $\bar{y}$ represent the predicted value, observed value, and average of observed data, respectively.

### 6.2 Quantitative results

The experiments on **Tables 2**–**4** demonstrate the effectiveness of the proposed PTCN model for weather forecasting at different time scales. The results show that PTCN32+ achieves the best or sub-optimal performance on most indicators, suggesting that the multi-scale feature representation provided by the pyramid temporal causal network is beneficial for the prediction accuracy. Moreover, the comparison between traditional and deep-learning-based methods indicates that the latter generally outperform the former for time series prediction tasks, such as weather forecasting.

**Table 2:** Comparison of proposed methods with some popular sequence prediction methods on WFD2018 aic (2018a).

Index	rh2m				t2m				w10m				
Matric	*RMSE*	*MAPE*	R^2^	*accuracy*	*RMSE*	*MAPE*	R^2^	*accuracy*	*RMSE*	*MAPE*	R^2^	*accuracy*	*AA*
WRF	17.91%	21.92%	0.40	78.40%	2.87°C	13.75%	0.60	88.38%	1.62m/s	96.03%	-1.11	33.08%	66.62%
GBR	28.48%	51.63%	-0.50	61.11%	6.64°C	33.27%	-0.89	70.37%	1.58m/s	82.53%	-0.55	40.54%	57.34%
LED	14.30%	22.27%	0.61	82.29%	4.36°C	21.08%	0.15	81.56%	1.17m/s	61.41%	0.17	56.47%	73.44%
LEDA	12.71%	19.60%	0.69	84.24%	4.31°C	20.93%	0.16	81.79%	1.19m/s	63.76%	0.14	55.62%	73.72%
GRU	12.49%	19.35%	0.71	84.90%	3.69°C	16.75%	0.37	84.83%	1.16m/s	59.55%	0.18	57.29%	75.67%
TCN8	10.79%	16.39%	0.78	87.42%	2.43°C	10.04%	0.74	90.64%	1.31m/s	72.53%	-0.04	50.11%	76.06%
TCN16	9.82%	14.30%	0.82	88.61%	2.10°C	8.81%	0.80	91.78%	1.15m/s	65.02%	0.16	55.12%	78.50%
TCN32	9.57%	13.91%	0.83	89.03%	2.05°C	8.32%	0.81	92.16%	1.09m/s	59.12%	0.25	58.28%	79.82%
PTCN8	10.16%	14.88%	0.81	88.26%	2.18°C	9.19%	0.78	91.44%	1.29m/s	71.03%	-0.02	50.44%	76.71%
PTCN8+	10.29%	15.28%	0.80	88.02%	2.19°C	9.15%	0.79	91.43%	1.07m/s	56.50%	0.27	59.43%	79.63%
PTCN32	9.70%	14.04%	0.82	88.91%	2.02°C	8.21%	0.82	92.33%	1.04m/s	54.50%	0.32	60.65%	80.63%
PTCN32+	9.69%	14.06%	0.83	88.94%	1.99°C	8.01%	0.82	92.48%	1.02m/s	52.59%	0.35	62.11%	81.18%

All experimental results are based on 32 hours of historical data to predict the next 6 hours. The larger the value of **R^2^**, accuracy and AA, the better, while the smaller the value of RMSE and MAPE, the better. The best results are labeled in red and the second best results in blue.

**Table 3:** Comparison of proposed methods with some popular sequence prediction methods on WFD2018 aic (2018a).

Index	rh2m				t2m				w10m				
Matric	*RMSE*	*MAPE*	*R^2^*	*accuracy*	*RMSE*	*MAPE*	*R^2^*	*accuracy*	*RMSE*	*MAPE*	*R^2^*	*accuracy*	*AA*
WRF	18.08%	21.89%	0.38	78.04%	2.76°C	13.83%	0.62	88.43%	1.64m/s	97.69%	-1.14	32.31%	66.26%
GBR	33.39%	63.16%	-1.07	54.17%	7.03°C	39.36%	-1.11	68.66%	1.62m/s	84.55%	-0.57	39.29%	54.04%
LED	15.26%	24.82%	0.57	80.08%	5.49°C	28.70%	-0.32	76.42%	1.26m/s	70.46%	0.04	52.22%	69.57%
LEDA	15.13%	24.56%	0.57	80.39%	4.71°C	24.56%	-0.02	79.79%	1.22m/s	67.00%	0.10	53.82%	71.33%
GRU	15.24%	22.60%	0.56	80.34%	4.11°C	21.17%	0.23	82.45%	1.23m/s	69.64%	0.09	53.15%	71.98%
TCN8	12.52%	19.23%	0.71	84.57%	2.63°C	11.58%	0.69	89.42%	1.38m/s	72.45%	-0.14	47.97%	73.99%
TCN32	11.60%	17.89%	0.75	85.90%	2.50°C	10.37%	0.72	90.34%	1.24m/s	66.95%	0.06	53.04%	76.43%
PTCN8	12.47%	18.88%	0.71	84.79%	2.59°C	11.18%	0.70	89.77%	1.33m/s	68.77%	-0.06	50.82%	75.13%
PTCN8+	12.32%	18.81%	0.71	84.93%	2.53°C	10.86%	0.71	90.23%	1.15m/s	58.69%	0.18	57.45%	77.54%
PTCN32	11.43%	16.95%	0.76	86.31%	2.26°C	9.34%	0.77	91.39%	1.13m/s	60.52%	0.21	57.62%	78.44%
PTCN32+	11.43%	16.85%	0.76	86.33%	2.25°C	9.23%	0.78	91.55%	1.09m/s	58.61%	0.27	59.24%	79.04%

All experimental results are based on 64 hours of historical data to predict the next 12 hours. Larger values of R^2^, accuracy, and AA indicate better performance, while smaller values of RMSE and MAPE indicate better performance. The best results are labeled in red and the second best results in blue.

**Table 4:** Comparison of proposed methods with popular sequence prediction methods on WFD2018 aic (2018a).

Index	rh2m				t2m				w10m				
Matric	*RMSE*	*MAPE*	*R^2^*	*accuracy*	*RMSE*	*MAPE*	*R^2^*	*accuracy*	*RMSE*	*MAPE*	*R^2^*	*accuracy*	*AA*
WRF	18.39%	22.08%	0.33	76.96%	2.63°C	14.29%	0.66	88.25%	1.63m/s	98.58%	-1.13	31.80%	65.67%
GBR	33.03%	64.58%	-1.18	53.05%	7.06°C	39.56%	-1.09	67.08%	1.62m/s	84.39%	-0.58	39.00%	53.04%
LED	17.52%	32.72%	0.34	76.53%	6.16°C	36.84%	-0.80	71.61%	1.27m/s	72.26%	0.03	51.38%	66.51%
LEDA	16.37%	32.57%	0.46	78.65%	5.78°C	34.58%	-0.45	74.63%	1.20m/s	63.99%	0.13	55.29%	69.52%
GRU	15.01%	27.12%	0.54	80.65%	5.21°C	30.22%	-0.31	76.50%	1.20m/s	65.68%	0.13	54.87%	70.67%
TCN8	13.98%	24.65%	0.61	81.41%	2.59°C	12.62%	0.69	88.78%	1.42m/s	80.43%	-0.21	45.11%	71.77%
TCN32	13.23%	23.86%	0.65	82.46%	2.28°C	11.10%	0.76	90.17%	1.23m/s	65.58%	0.09	53.58%	75.40%
PTCN8	13.85%	24.65%	0.61	81.68%	2.47°C	11.73%	0.72	89.24%	1.30m/s	69.11%	-0.01	50.67%	73.86%
PTCN8+	13.74%	24.36%	0.62	81.95%	2.35°C	11.56%	0.74	89.61%	1.18m/s	65.03%	0.15	54.87%	75.48%
PTCN32	13.31%	23.63%	0.64	82.60%	2.31°C	10.78%	0.76	90.18%	1.20m/s	63.80%	0.11	54.62%	75.80%
PTCN32+	13.25%	23.69%	0.65	82.68%	2.20°C	10.38%	0.79	90.61%	1.15m/s	61.29%	0.19	56.70%	76.66%

All experimental results are based on 128 hours of historical data to predict the next 24 hours. Larger values of R^2^, accuracy and AA indicate better performance, while smaller values of RMSE and MAPE indicate better performance. The best results are labeled in red and the second best results in blue.

It is also observed that the prediction accuracy decreases as the length of the predicted weather information increases. This could be due to the increasing complexity of the task, as longer-term predictions require the model to capture more complex and subtle patterns in the weather data. However, PTCN still shows promising performance even for longer-term predictions.

Overall, the PTCN model’s ability to learn weather periodicity information from different scales and combine them effectively is a key factor in achieving its superior performance. Additionally, the almost similar number of parameters between PTCN32 and TCN32 shows that using multi-scale features does not significantly increase the model’s complexity, but can lead to better results.

In addition, it is worth noting that the proposed method outperforms the other deep learning models, such as LED and LEDA, which are based on attention mechanisms. This suggests that the multi-scale feature representation in PTCN is more effective than the attention mechanism for weather forecasting. It is also worth mentioning that the results of the PTCN model are competitive with those of WRF, a widely used numerical weather prediction model, especially for shorter forecasting horizons. This suggests that deep learning-based methods such as PTCN have great potential in weather forecasting applications.

Furthermore, the experiments show that the longer the forecasting horizon, the less accurate the prediction, which is consistent with the general characteristics of weather prediction. The PTCN model can provide accurate predictions for up to 12 hours in advance, but its performance decreases significantly when predicting 24 hours in advance. This highlights the challenges of long-term weather prediction and the need for further research in this area.

#### 6.2.1 Validation of pyramid temporal causal network

The validation of the Pyramid Temporal Causal Network (PTCN) demonstrates the effectiveness of utilizing multi-scale feature representation for sequence prediction tasks. The comparison between TCN32 and PTCN32, and between TCN32+ and PTCN32+, shows that PTCN models consistently achieve better results on most indicators. This suggests that the inclusion of multi-scale features through the pyramid structure of the PTCN model allows for more powerful predictive capabilities than the traditional TCN model.

Furthermore, the small increase in FLOPs and parameters required for using multi-scale features in the PTCN model highlights its efficiency. This efficiency is especially notable when compared to the significant improvements in the prediction accuracy of the AA indicator and most other indicators. For example, the increase in the AA index by 1.69% achieved by PTCN16 compared to TCN16, while only increasing the FLOPs and parameters by 0.74M and 2.88K, respectively, is a strong indicator of the benefits of multi-scale feature representation.

Overall, the validation of the PTCN model emphasizes the importance of considering multi-scale features for sequence prediction tasks. By utilizing different time scale feature information through a pyramid temporal causal network, the PTCN model can approximate the ground truth by linearly fitting multiple scale features. This approach allows for better prediction accuracy and efficiency with almost the same amount of parameters.

#### 6.2.2 Validation of MVLoss

The MVLoss function is a new loss function proposed in this work to handle multivariate time series prediction problems with variables of different variances. To use the MVLoss loss function, the global variance of each variable that needs to be predicted is first calculated. Then, the MVLoss function takes into account the variance of each variable in the loss calculation.

To use the MVLoss loss function, we first need to calculate the global variance of each variable that needs to be predicted. The global variances of the following three variables are:

- 2 meters temperature (t2m): 149.11
- 2 meters relative humidity (rh2m): 657.15
- 10 meters wind speed (w10m): 2.67

**Table 5** compares the performance of the models optimized with MVLoss and MSE loss functions, respectively. The models with MVLoss have better results in the AA indicator than those with the MSE loss function, and they can significantly improve the prediction accuracy of small variance variables. For example, the prediction accuracy of TCN32+^(6)^ on w10m is much higher than that of TCN32^(6)^ by 4.32%. This demonstrates that the proposed MVLoss can improve the accuracy of small variance variables. However, the prediction results of variables with large variance tend to be reduced, but the degree of change is small.

**Table 5:** Ablation experiments.

Index	rh2m				t2m				w10m				
Matric	*RMSE*	*MAPE*	*R^2^*	*accuracy*	*RMSE*	*MAPE*	*R^2^*	*accuracy*	*RMSE*	*MAPE*	*R^2^*	*accuracy*	*AA*
TCN32^(6)^	9.57%	13.91%	0.83	89.03%	2.05°C	8.32%	0.81	92.16%	1.09m/s	59.12%	0.25	58.28%	79.82%
PTCN32^(6)^	9.70%	14.04%	0.82	88.91%	2.02°C	8.21%	0.82	92.33%	1.04m/s	54.50%	0.32	60.65%	80.63%
TCN32+^(6)^	9.65%	13.99%	0.83	88.89%	2.08°C	8.29%	0.81	92.15%	1.00m/s	52.22%	0.37	62.60%	81.21%
PTCN32+^(6)^	9.69%	14.06%	0.83	88.94%	1.99°C	8.01%	0.82	92.48%	1.02m/s	52.59%	0.35	62.11%	81.18%
TCN32^(12)^	11.60%	17.89%	0.75	85.90%	2.50°C	10.37%	0.72	90.34%	1.24m/s	66.95%	0.06	53.04%	76.43%
PTCN32^(12)^	11.43%	16.95%	0.76	86.31%	2.26°C	9.34%	0.77	91.39%	1.13m/s	60.52%	0.21	57.62%	78.44%
TCN32+^(12)^	11.56%	17.89%	0.75	86.01%	2.55°C	10.59%	0.71	90.13%	1.12m/s	59.96%	0.23	58.26%	78.13%
PTCN32+^(12)^	11.43%	16.85%	0.76	86.33%	2.25°C	9.23%	0.78	91.55%	1.09m/s	58.61%	0.27	59.24%	79.04%
TCN32^(24)^	13.23%	23.86%	0.65	82.46%	2.28°C	11.10%	0.76	90.17%	1.23m/s	65.58%	0.09	53.58%	75.40%
PTCN32^(24)^	13.31%	23.63%	0.64	82.60%	2.31°C	10.78%	0.76	90.18%	1.20m/s	63.80%	0.11	54.62%	75.80%
TCN32+^(24)^	13.29%	24.06%	0.64	82.49%	2.20°C	10.49%	0.78	90.62%	1.16m/s	63.67%	0.18	56.09%	76.40%
PTCN32+^(24)^	13.25%	23.69%	0.65	82.68%	2.20°C	10.38%	0.79	90.61%	1.15m/s	61.29%	0.19	56.70%	76.66%

The larger the value of **R^2^**, accuracy and AA, the better, and the smaller the value of RMSE and MAPE, the better. The superscripts ^(6)(12)(24)^ indicate that the model uses 32, 64 or 128 hours of historical weather information to predict the future 6, 12 or 24 hours of weather information, respectively.

It is worth noting that the MVLoss function is more suitable for multivariate prediction models than the MSE loss function from the analysis of the overall prediction accuracy AA index. This is because the MVLoss function can effectively deal with variables with different variances, which can lead to a more accurate prediction of the overall AA index.

Overall, the MVLoss function provides a better solution for handling multivariate time series prediction problems with variables of different variances, which is a common issue in many real-world applications.

### 6.3 Qualitative discussions

In this section, we will qualitatively analyze the changes in loss during training and testing, and compare the prediction results of the PTCN32+ with those of WRF.

#### 6.3.1 Change in loss

**Figure 4:**
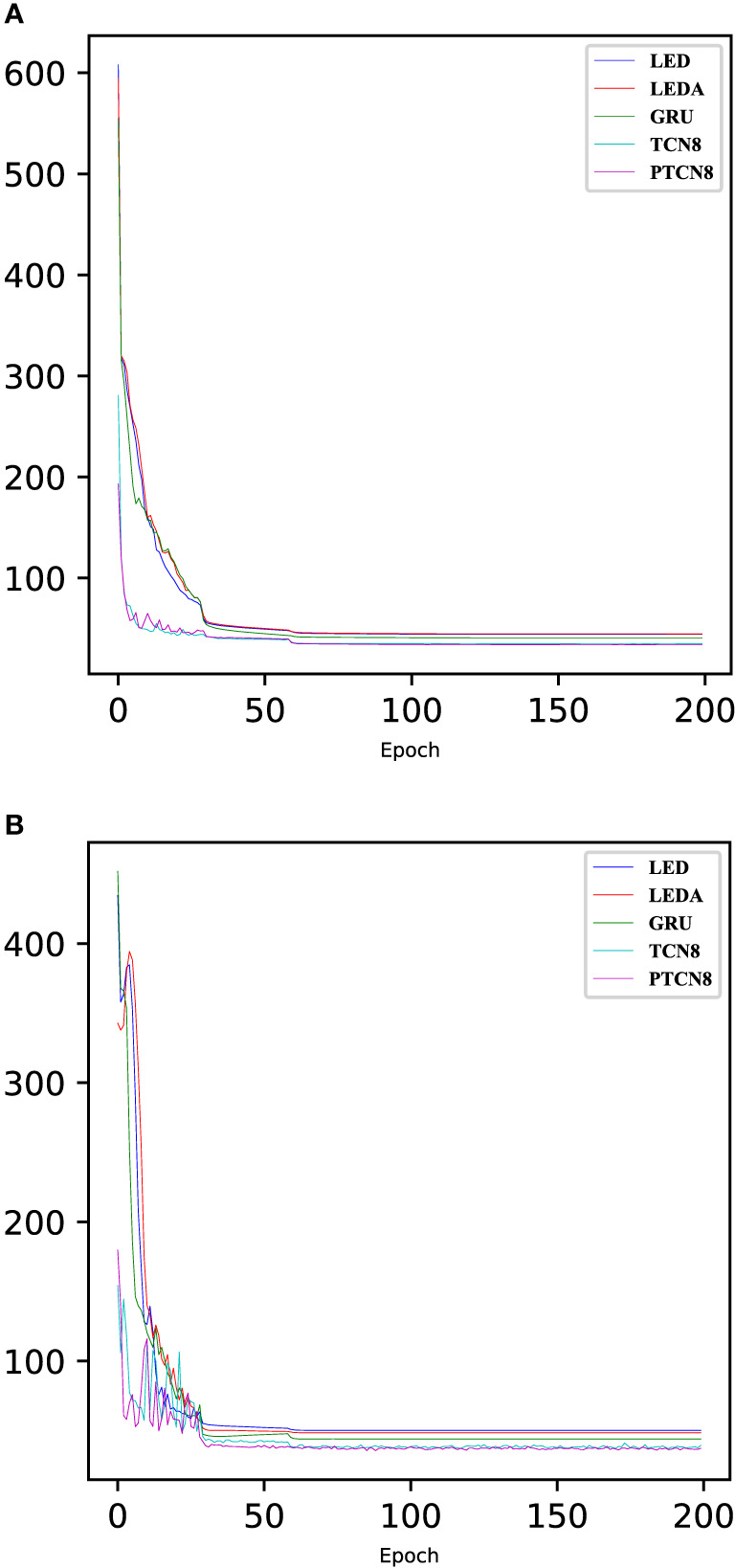
The training and testing losses when predict the next 6 hours in station 2.

**Figure 4** shows the changes in loss during model training and testing. As can be seen from the figure, the PTCN8 model achieves the smallest loss during both training and testing. Furthermore, TCN8 and PTCN8 converge faster than other methods. It can be qualitatively seen that the proposed model has better predictive accuracy than all compared methods.

#### 6.3.2 Forecast result analysis

**Figure 5:**
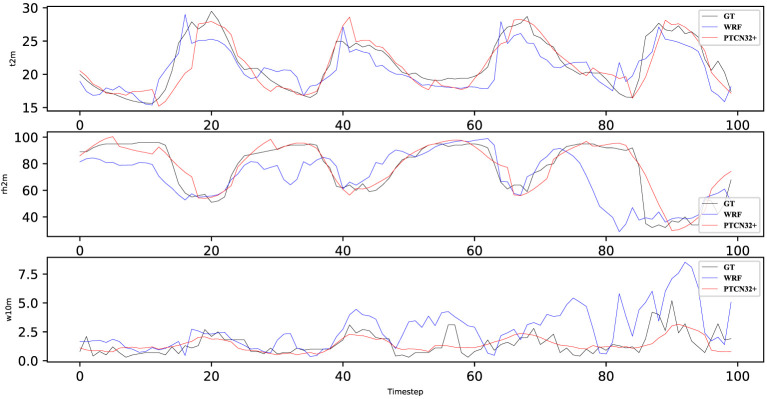
Weather predicted result when predict the next 6 hours in station 2.

In **Figure 5**, we can see that the PTCN32+ model predicts all three meteorological factors (rh2m, t2m, and w10m) closer to the ground truth (GT) than the WRF model. This suggests that the PTCN32+ model can improve the prediction accuracy compared to the commonly used WRF model. Specifically, the PTCN32+ model predicts rh2m and t2m more accurately than WRF, with smaller deviations from the GT. For the w10m variable, although both models have similar accuracy, PTCN32+ still shows a slight advantage in terms of predicting higher wind speeds.

The improved prediction accuracy of the PTCN32+ model can be attributed to its ability to capture complex temporal relationships and features in the input data, which is not possible in traditional physical modeling methods like WRF. Moreover, the proposed temporal attention mechanism enables the model to selectively focus on important time steps, further improving the accuracy of the predictions. These results demonstrate the potential of deep learning models, specifically the PTCN32+ model, to improve weather forecasting accuracy and provide more reliable predictions for applications in various industries.

## 7 Conclusion

In this paper, we proposed the PTCN model for weather forecasting, which can use different time-scale features and improve the weather forecasting results. We argued that this is because features at different scales may imply different patterns of variation that are important for weather prediction. Additionally, we proposed a loss function that normalizes the variables in a multivariate prediction task, which can help improve the effectiveness of temporal prediction models for predicting multiple variables simultaneously. Through experiments and analysis, we demonstrated that the PTCN model outperforms several state-of-the-art methods in meteorological forecasting, and we believe that it has great potential for application in the field of weather forecasting.

## Data availability statement

The dataset will be available at the following website: https://github.com/YuanMLer/AI-Challenger-Global-AI-Contest-.

## Author contributions

The author confirms being the sole contributor of this work and has approved it for publication.
